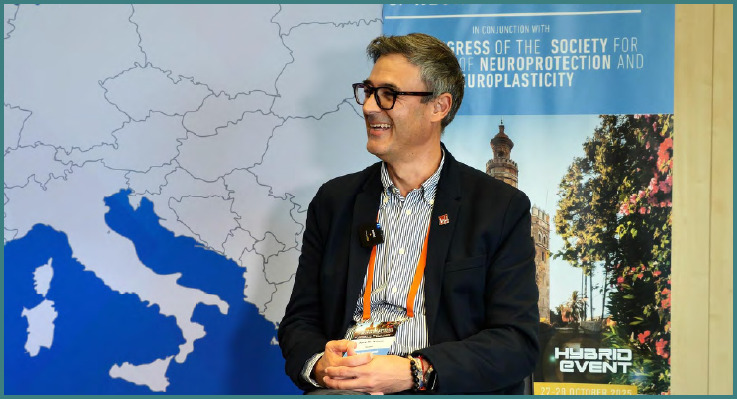# Interview with Prof. José M. Azorín - 8^th^ European Congress on Neurorehabilitation in conjunction with the 20^th^ Congress of the Society for the Study of Neuroprotection and Neuroplasticity

**DOI:** 10.25122/jml-2026-1005

**Published:** 2026-03

**Authors:** Stefana-Andrada Dobran

**Affiliations:** 1 RoNeuro Institute for Neurological Research and Diagnostic, Cluj-Napoca, Romania; 2 Sociology Department, Babes-Bolyai University, Cluj-Napoca, Romania

**Interviewee: Professor José M. Azorín**

**Interviewer: Ms. Stefana-Andrada Dobran**

Professor José M. Azorín is a leading figure in neurotechnology and Brain-Machine Interfaces (BMIs). As the Director of the Brain-Machine Interface Systems Lab and Deputy Vice-Rector of Research at Miguel Hernández University of Elche (Spain), he spearheads innovative research using BMIs based on artificial intelligence (AI) to control robotic exoskeletons for rehabilitation. He also directs the only European site of the prestigious BRAIN (Building Reliable Advances and Innovation in Neurotechnology) Center. Prof. Azorín was also visiting professor at the University of Houston (USA) and at Imperial College London (United Kingdom) and has received several prestigious grants from the European Union for his research projects. Currently, he is president of AITADIS (Iberoamerican Society of Assistive Technologies) and he is a Distinguished Lecturer of the IEEE Systems Council.

**
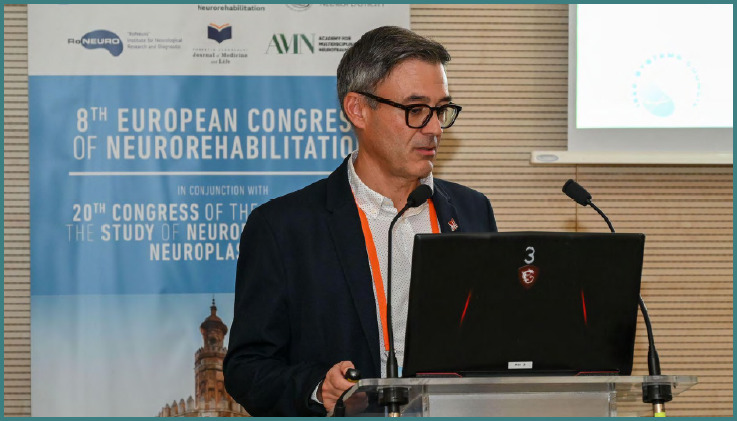
**

**S.D.: Hello, Professor José Azorín, and welcome to the 8^th^ European Congress of Neurorehabilitation in conjunction with the 20^th^ Congress of the Society for the Study of Neuroprotection and Neuroplasticity. This European Congress brings together the scientific and clinical communities. What do you believe is the unique role it plays in bridging the gap between research and daily patient care in neurorehabilitation?**

J.A.: Hello, it's a pleasure to be here in this conference. I'm a full professor, however, my background is not in clinical medicine. In my case, I think that this conference is the best scenario to try to power this interaction between clinician and technical people. Because in the end, we have to cooperate if we want to advance the field.

**S.D.: Considering your specialty, what future developments do you envision for the complex multidisciplinary field of neurorehabilitation?**

J.A.: In my case, I am very focused on motor rehabilitation. Currently, what we are doing is decoding brain signals to try to command external devices, such as exoskeletons for walking or for helping people move their arms. I think that right now, artificial intelligence is growing very fast, and we can take advantage of that if we are able to decode brain signals more effectively. If we understand the brain better, I believe we will be able to achieve more significant advances in motor rehabilitation.

**S.D.: What emerging trend or technology from this field are you most excited about and looking forward to?**

J.A.: I think in the future we will need to evolve the hardware system, because we are still recording signals in the same way as we were many years ago. New materials could certainly advance the field. But of course, I am really interested in non-invasive brain-computer interfaces. We can use invasive technology, but I think we should focus on improving the decoding of non-invasive brain signals... decoding brain signals–specifically, electroencephalography (EEG) signals–and I'm sure we can do that if we begin applying new artificial learning techniques.

**S.D.: What is, in your perspective, the most challenging future development in neurorehabilitation and how can EFNR come close to this endeavor?**

J.A.: On the one hand, I think we need to move toward low-cost devices or systems, because currently, when a person needs motor rehabilitation, they often have to use large exoskeletons that are expensive. Even the devices for decoding EEG signals are costly. So, I believe we must shift toward more affordable rehabilitation solutions that people can use at home. Otherwise, many individuals simply cannot afford this technology.

Until now, the focus has been on hospital or clinical settings, but we need to advance further. We have to develop low-cost systems and transfer this technology directly to the people who need it.

**S.D.: Considering your research experience on brain machine interfaces, what is one key insight from your work that has impacted your understanding on how the brain interacts with machines to regain movement?**

J.A.: When I started to research in this field, I was thinking about *brain-computer interfaces*, as an independent system that we calibrated in order to decode brain signals. However, I have discovered that this is not really true. A *brain-computer interface* is like a symbiotic system. Humans adjust to the *brain-computer interface*; the person has to adjust generating the brain signals in order to command some devices. At the same time, we are calibrating the brain-machine interface to get used to the brain signal of the user. So, as a result of this collaboration–of this symbiotic behavior of the system–we are able to command devices from brain activity.